# Supplementary material for: Shorebirds-driven trophic cascade helps restore coastal wetland multifunctionality
Source: Nat Commun. 2023 Dec 6;14:8076. doi: 10.1038/s41467-023-43951-3 (PMC10700615; doi:10.1038/s41467-023-43951-3)
Supplement: Supplementary file 1 — Supplementary Information [file 41467_2023_43951_MOESM1_ESM.pdf]

## Supplementary Information for

# Shorebirds-driven trophic cascade helps restore coastal wetland multifunctionality

Chunming Li, Jianshe Chen, Xiaolin Liao, Aaron P. Ramus, Christine Angelini, Lingli Liu, Brian R. Silliman, Mark D. Bertness & Qiang He*

* Corresponding author. Email address: he_qiang@hotmail.com

2017).

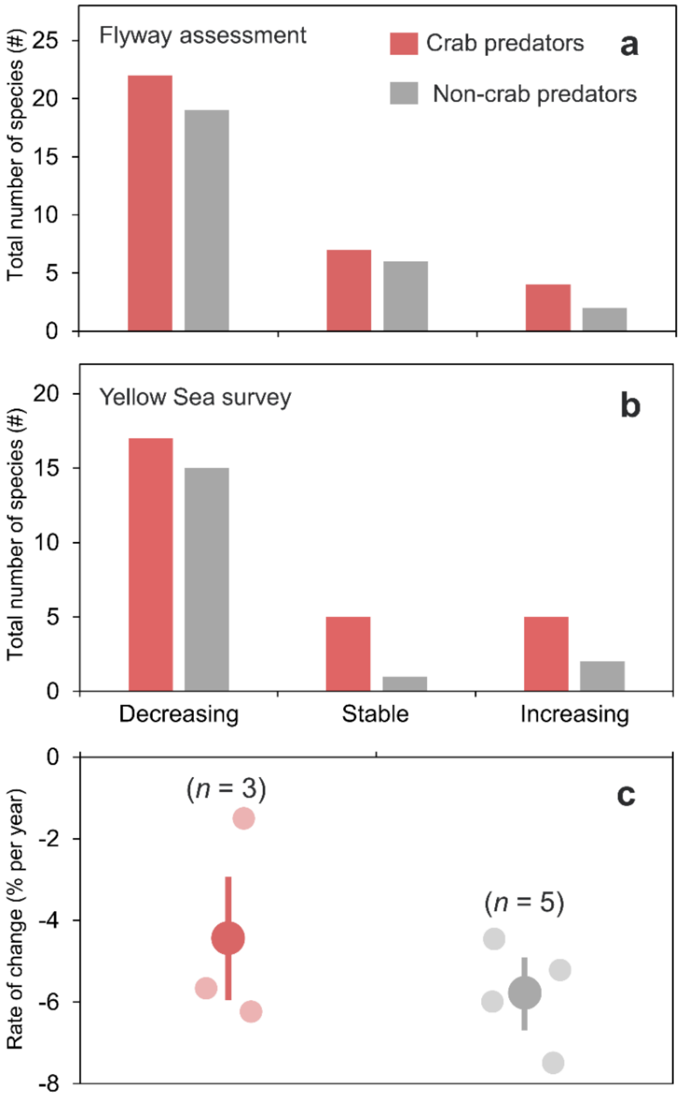


**Supplementary Fig. 1: Population trends of shorebird species that consume and do not consume crabs.** (**a**) Flyway assessment based on assessments of the global population of all shorebird species that utilize coastal habitats in the Yellow Sea. (**b**) Yellow Sea survey. Given in **a** and **b** are the numbers of shorebird species with a decreasing, stable, and increasing population. (**c**) Annual rate of change in the population size of shorebirds with a strong reliance on coastal habitats in the Yellow Sea (*n* indicates independent species; data from Studds *et al.* 2017).

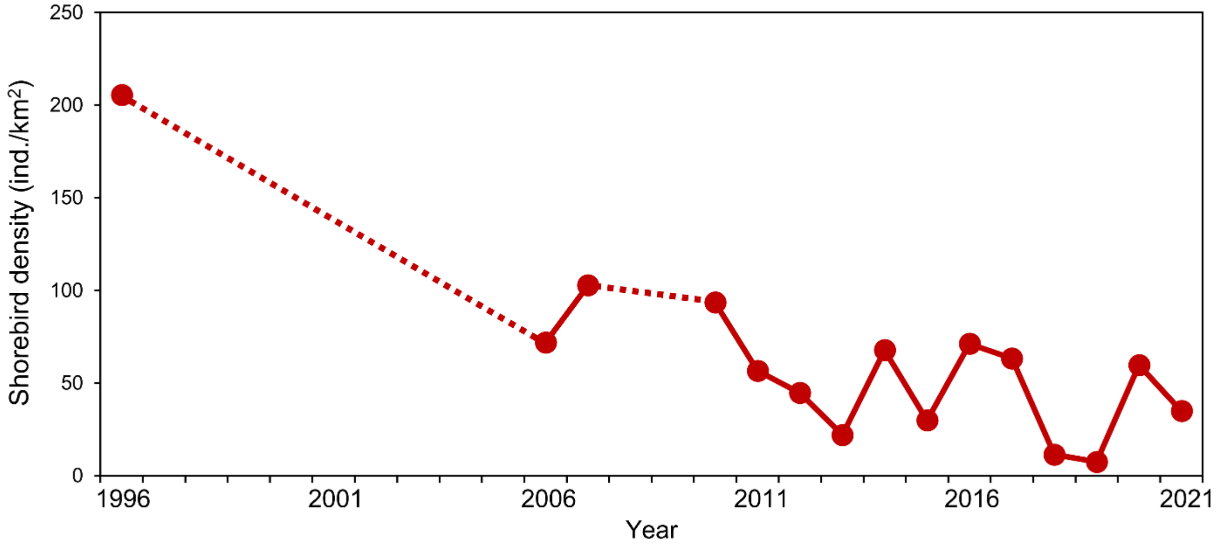


**Supplementary Fig. 2: Long-term trend in shorebird density during northward migration.** The shorebird surveys were conducted in the intertidal zones of Shanghai Chongming Dongtan National Nature Reserve.

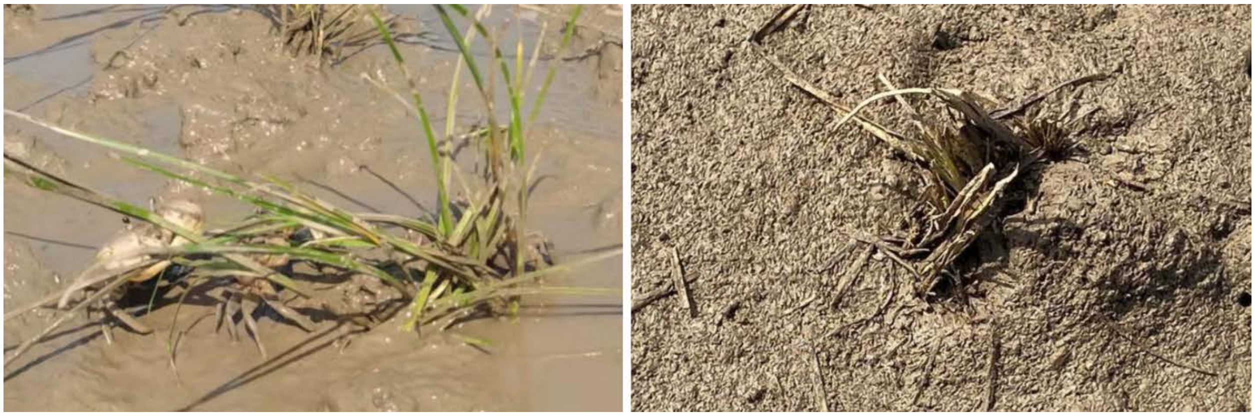

**Supplementary Fig. 3: Photographs showing crab grazing on aboveground stems of planted *Scirpus*.** Photo credit: Chunming Li. Also, see Supplementary Movie 1.

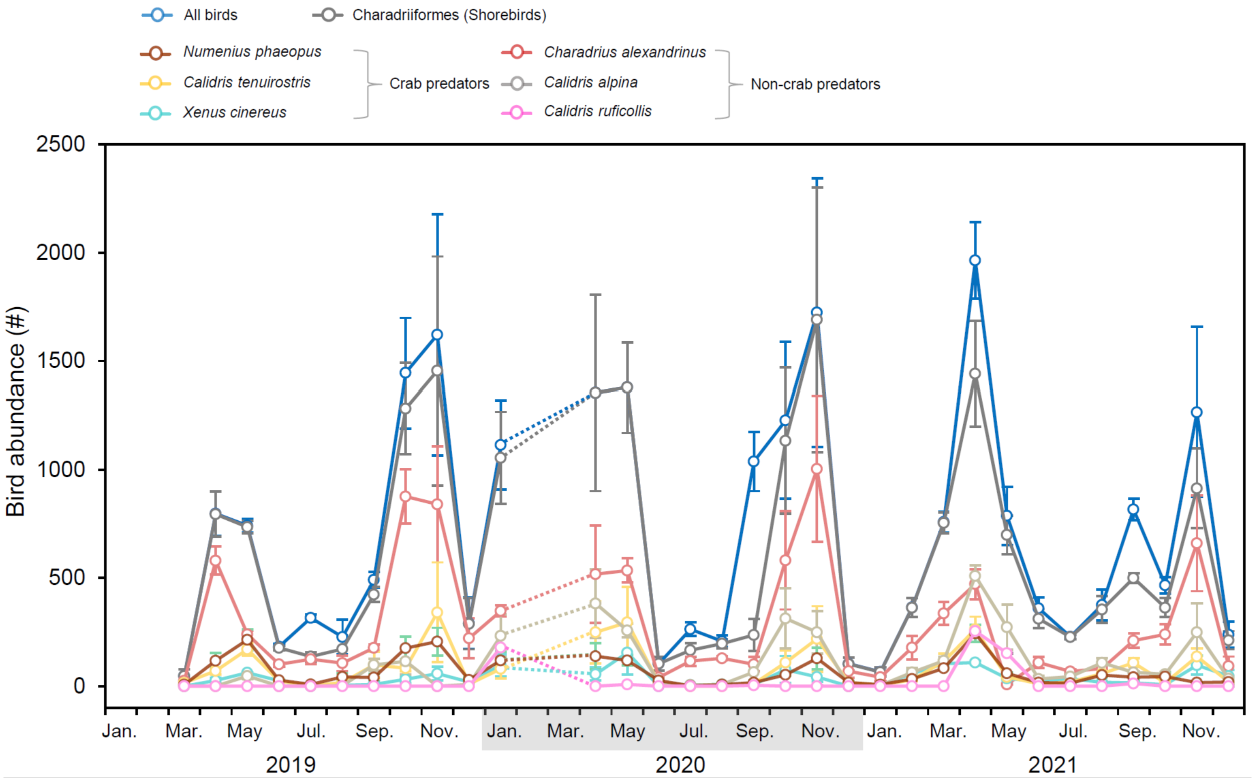


**Supplementary Fig. 4: Monthly changes in shorebird abundance at the restoration site from 2019 to 2021.** The total abundance of all birds, all shorebirds, as well as the abundance of six shorebird species with the highest total abundance, are shown. Data are shown as means ± SE ($n$ = 3 independent camera traps).

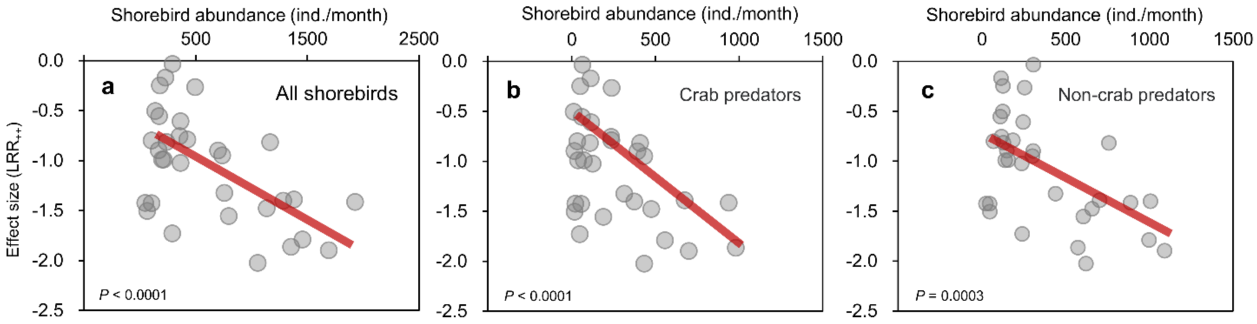


**Supplementary Fig. 5: Variation in the effect size of shorebirds on crab abundance against shorebird abundance:** (**a**) All shorebirds, (**b**) crab predators, and (**c**) non-crab predators. Red lines indicate a statistically significant meta-regression ($P < 0.05$; see Supplementary Table 3 for test statistics). For all panels, $n = 32$ repeated measurements (potential autocorrelation was accounted for by including plot ID as a random effect; see Methods).

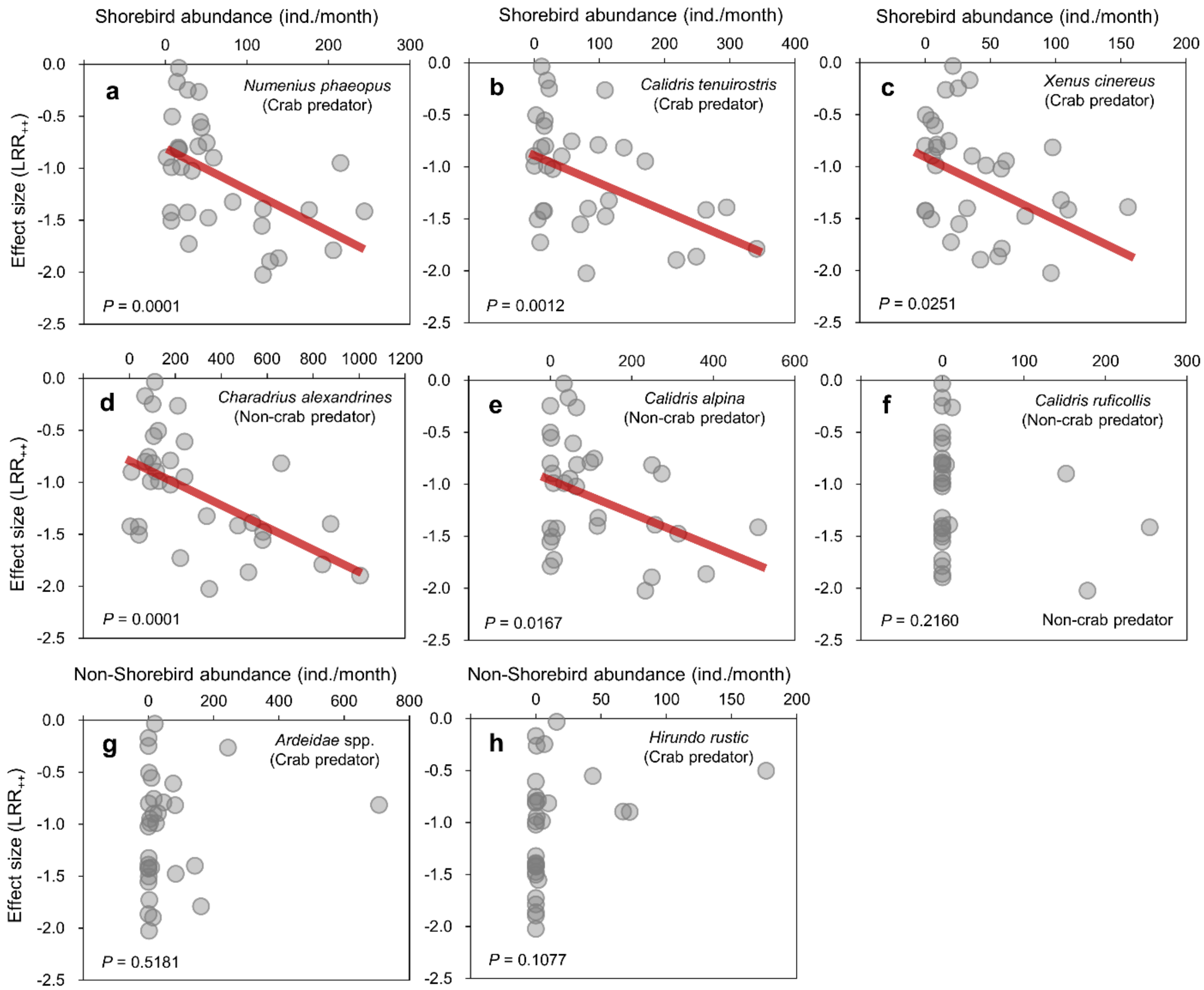


**Supplementary Fig. 6: Variation in the effect size of birds on crab abundance against bird abundance.** (**a**) *Numenius phaeopus*, (**b**) *Calidris tenuirostris*, (**c**) *Xenus cinereus*, (**d**) *Charadrius alexandrines*, (**e**) *Calidris alpina*, (**f**) *Calidris ruficollis*, (**g**) *Ardeidae spp.*, and (**h**) *Hirundo rustic*. Red lines indicate a statistically significant meta-regression ($P < 0.05$; see Supplementary Table 3 for test statistics). For all panels, $n = 32$ repeated measurements (potential autocorrelation was accounted for by including plot ID as a random effect; see Methods).

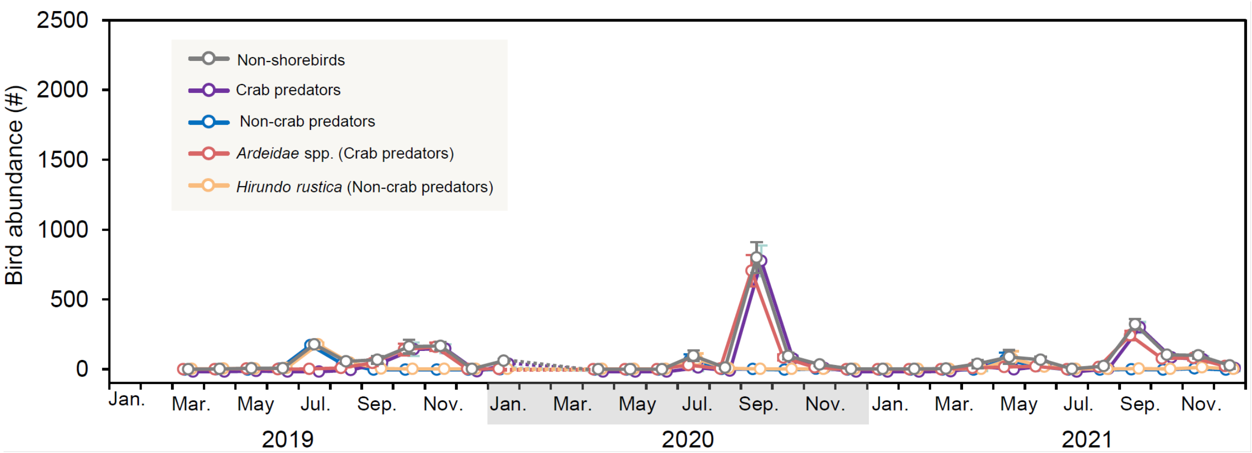


**Supplementary Fig. 7: Trends in the abundances of non-shorebirds at the study site from 2019 to 2021.** Non-shorebirds are further categorized into crab predators and non-crab predators. Two non-shorebird species with the highest total abundance are also shown. Data are shown as means with error bars for standard errors (*n* = 3 independent camera traps).

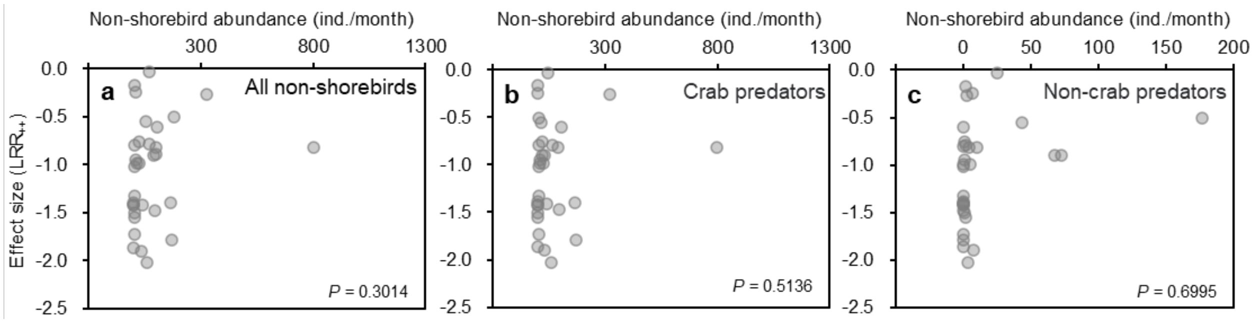


**Supplementary Fig. 8: Variation in the effect size of birds on crab abundance against the abundance of non-shorebirds.** (**a**) All non-shorebirds; (**b**) crab predators, and (**c**) non-crab predators. None of the meta-regression models were statistically significant (see Supplementary Table 3 for test statistics). For all panels, *n* = 32 repeated measurements (potential autocorrelation was accounted for by including plot ID as a random effect; see Methods).

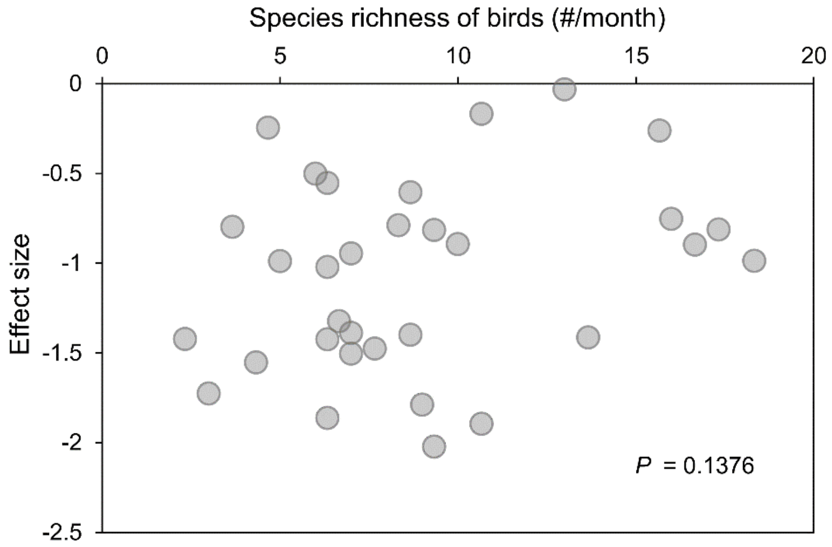


**Supplementary Fig. 9: Variation in the effect size of birds on crab abundance against the species richness of birds.** The meta-regression model was not statistically significant (*n* =32 repeated measurements; potential autocorrelation was accounted for by including plot ID as a random effect; see Supplementary Table 3 for test statistics).

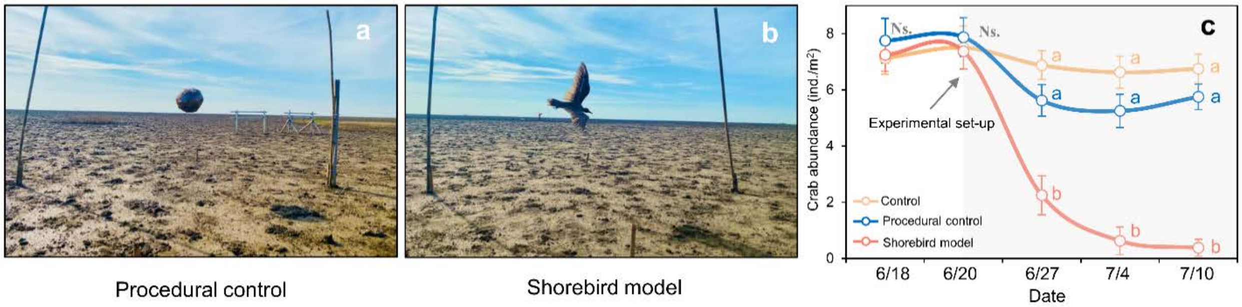


**Supplementary Fig. 10: Non-consumptive effects of shorebirds on crab grazers.** (**a**) Procedural control using a ball with similar size and color. (**b**) Simulation of the consumptive effects of shorebirds using a swinging shorebird (*Numenius phaeopus*) model. (**c**) Crab abundance in different treatments before and after the experimental set-up (one-way ANOVA; see Supplementary Table 4 for test statistics). Data are shown as means ± SE ($n = 8$ independent plots). Of a sampling date, points that do not share a letter differ significantly from one another based on Tukey's HSD multiple comparisons ($P < 0.05$; detailed statistical results are provided in Supplementary Table 5).

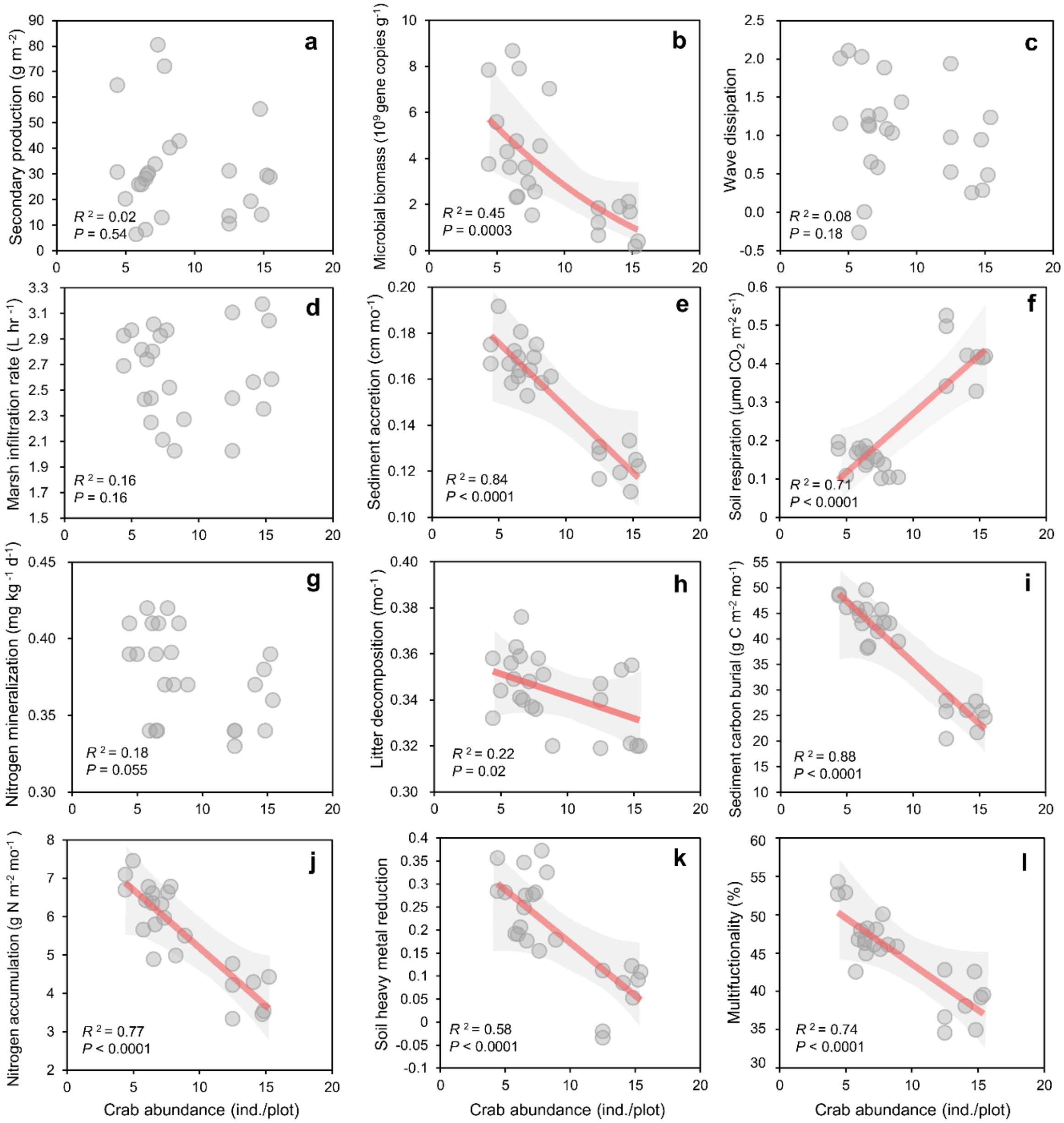


**Supplementary Fig. 11: Individual ecosystem functions and average multifunctionality as a function of crab abundance.** Red lines indicate a statistically significant linear or quadratic regression ($P < 0.05$; see Supplementary Table 9 for test statistics). Shaded areas are 95% confidence intervals ($n = 24$ independent plots).

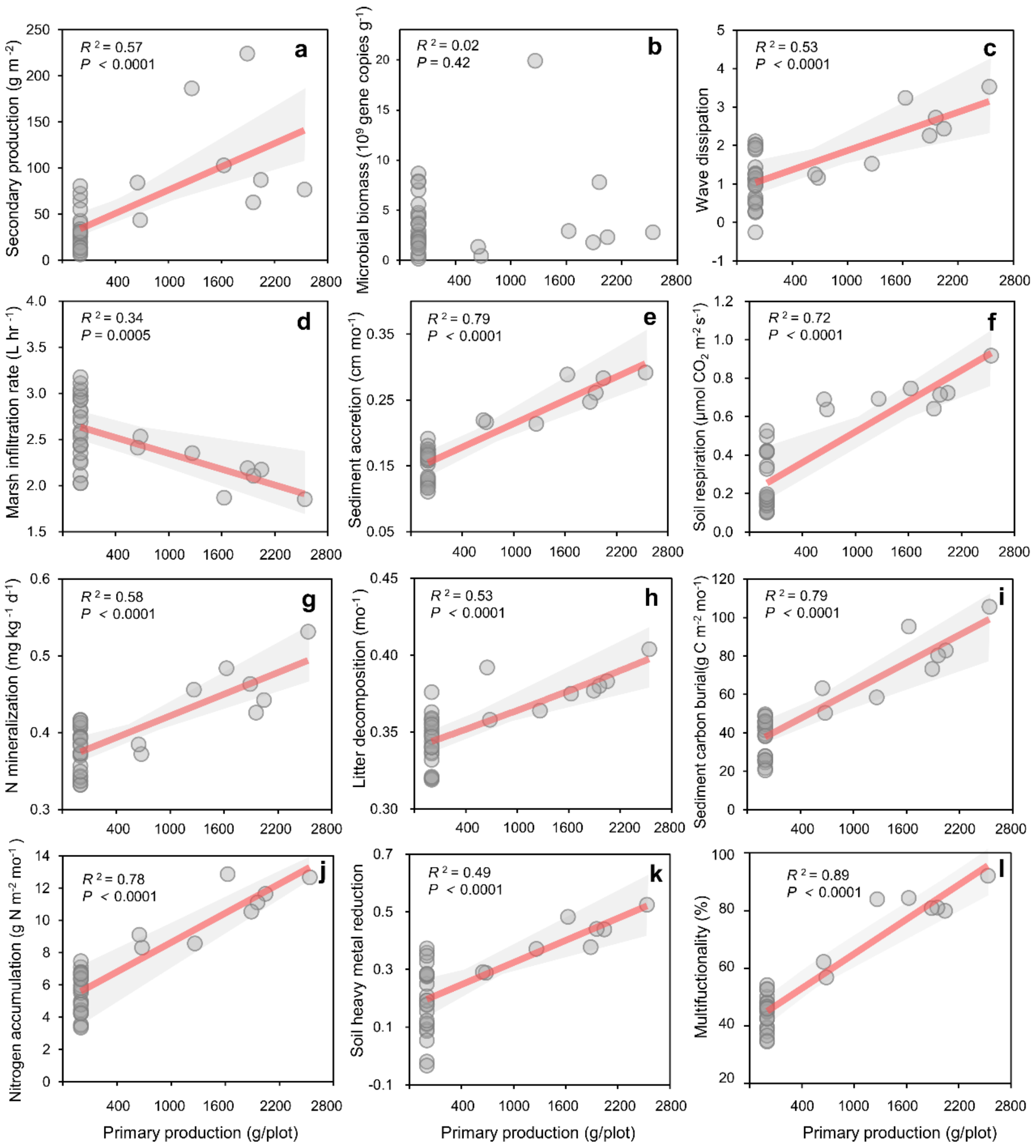


**Supplementary Fig. 12: Individual ecosystem functions and average multifunctionality as a function of primary production.** Red lines indicate a statistically significant linear or quadratic regression ($P < 0.05$; see Supplementary Table 10 for test statistics). Shaded areas are 95% confidence intervals ($n$ = 32 independent plots).

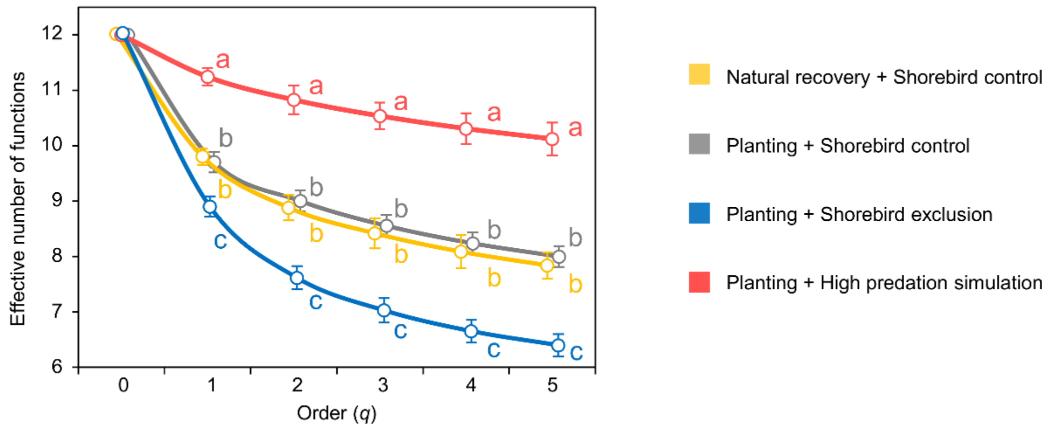


**Supplementary Fig. 13: The effective number of functions in different restoration treatments.** The effective number of functions is the equivalent number of functions were all functions provided at the same level (Byrnes *et al.* 2023). The effective number of functions is just the number of functions measured when order $q = 0$ and analogue to Shannon diversity for species when $q = 1$. When $q > 1$, functions performing at higher levels are given greater weight. Data are shown as means with error bars for standard errors ($n = 8$ independent plots, one-way ANOVA; see Supplementary Table 12 for test statistics). Treatments that do not share a letter differ significantly from one another based on Tukey HSD multiple comparisons ($P < 0.05$; detailed statistical results are provided in Supplementary Table 13).

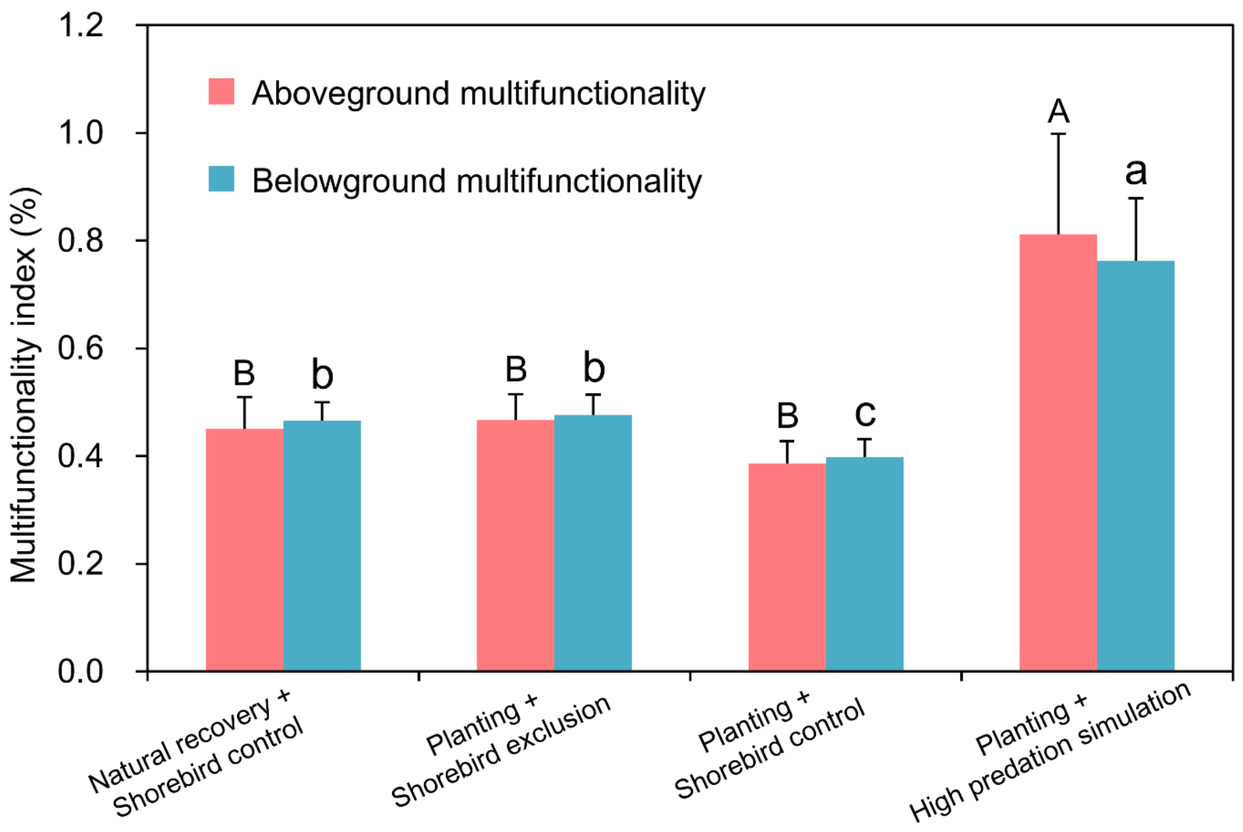


**Supplementary Fig. 14: Below- and aboveground multifunctionality in different restoration treatments.** Data are shown as means with error bars for standard errors ($n$ = 8 independent plots). Within a group of belowground multifunctionality (one-way ANOVA, df = 3, 28, $F$ = 27.76, $P < 0.0001$) and aboveground multifunctionality (one-way ANOVA, df = 3, 28, $F$ = 55.5, $P < 0.0001$), bars sharing a letter are not significantly different from one another based on Tukey's HSD multiple comparisons ($P < 0.05$; detailed statistical results are provided in Supplementary Table 16).

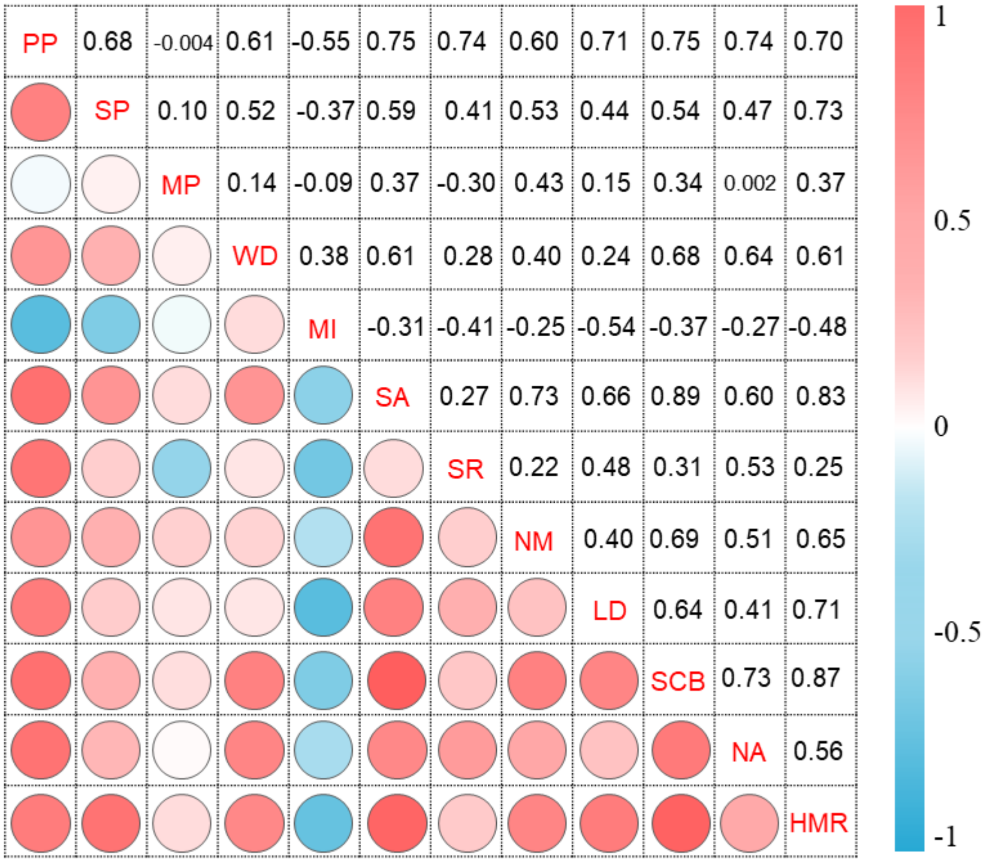


**Supplementary Fig. 15: Correlation between the twelve ecosystem functions.** PP: primary production of *Scirpus mariqueter*, SP: secondary production, MP: microbial production, WD: wave dissipation, MI: marsh infiltration, SA: sediment accretion, SR: soil respiration, NM: nitrogen mineralization, LD: litter decomposition, SCB: sediment carbon burial, NA: nitrogen accumulation, and HMR: soil heavy metal reduction.

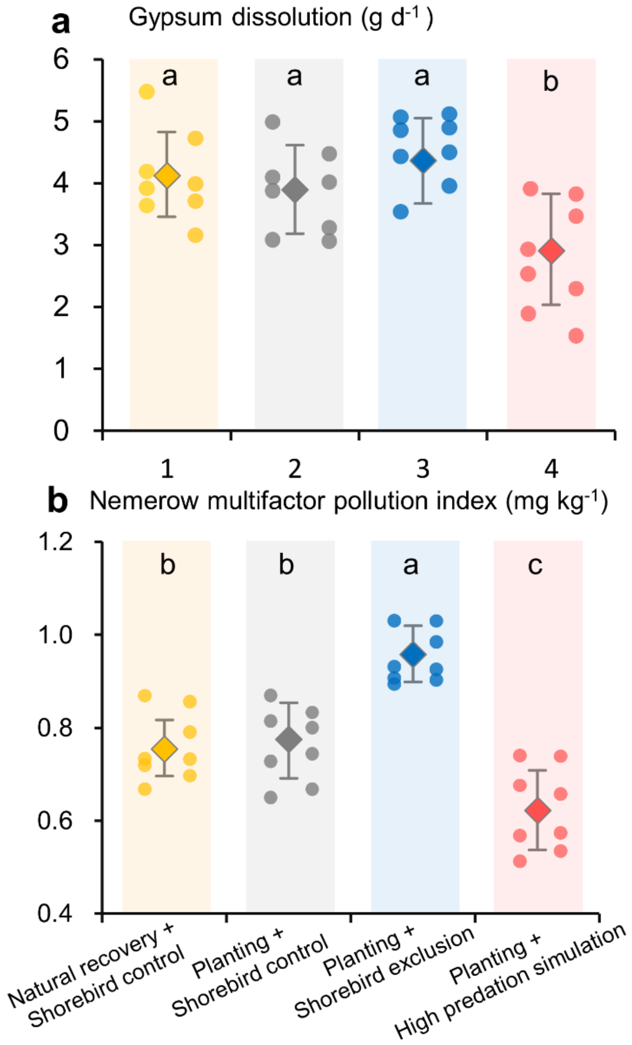


**Supplementary Fig. 16: Individual ecosystem functions in different restoration treatments.** (**a**) gypsum dissolution (one-way ANOVA; df = 3, 28, $F = 6.101$, $P = 0.0025$), and (**b**) Nemerow multifactor pollution index (one-way ANOVA; df = 3, 28, $F = 6.101$, $P < 0.0001$). Data are shown as means with error bars for standard errors ($n = 8$ independent plots). Bars sharing a letter are not significantly different from one another based on Tukey's HSD multiple comparisons ($P < 0.05$; detailed statistical results are provided in Supplementary Table 19).

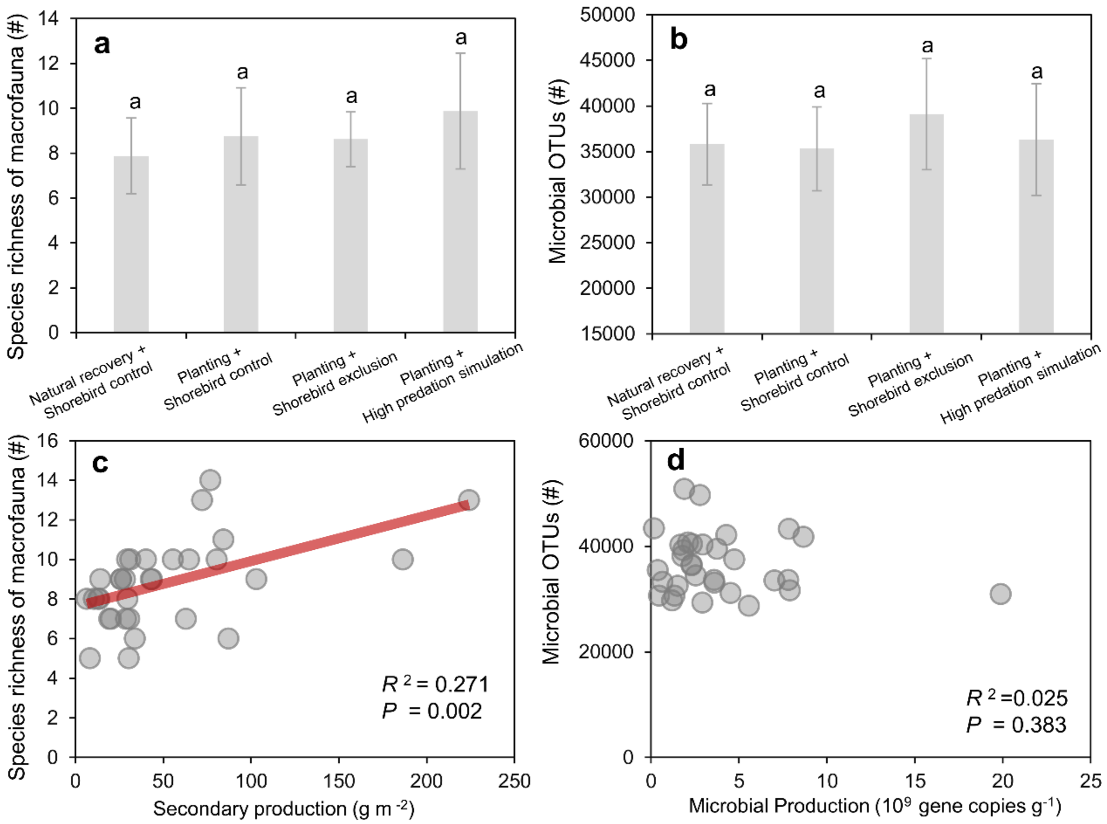


**Supplementary Fig. 17: Treatment effects on macrofauna and microbial diversity.** (**a**) Species richness of macrofauna (one-way ANOVA; df = 3, 28, $F$ = 1.2191, $P$ = 0.3211), (**b**) microbial operational taxonomic units (OTUs) (one-way ANOVA; df = 3, 28, $F$ = 0.6984, $P$ = 0.5609), (**c**) species richness of macrofauna as a function of macrofauna biomass, and (**d**) microbial OTUs as a function of microbial production. In **a** and **b**, data are shown as means with error bars for standard errors ($n$ = 8 independent plots). Bars sharing a letter are not significantly different from one another based on Tukey's HSD multiple comparisons ($P$ < 0.05; detailed statistical results are provided in Supplementary Table 20). In **c** and **d**, red lines indicate a statistically significant regression ($n$ = 32 independent plots; $P$ < 0.05).

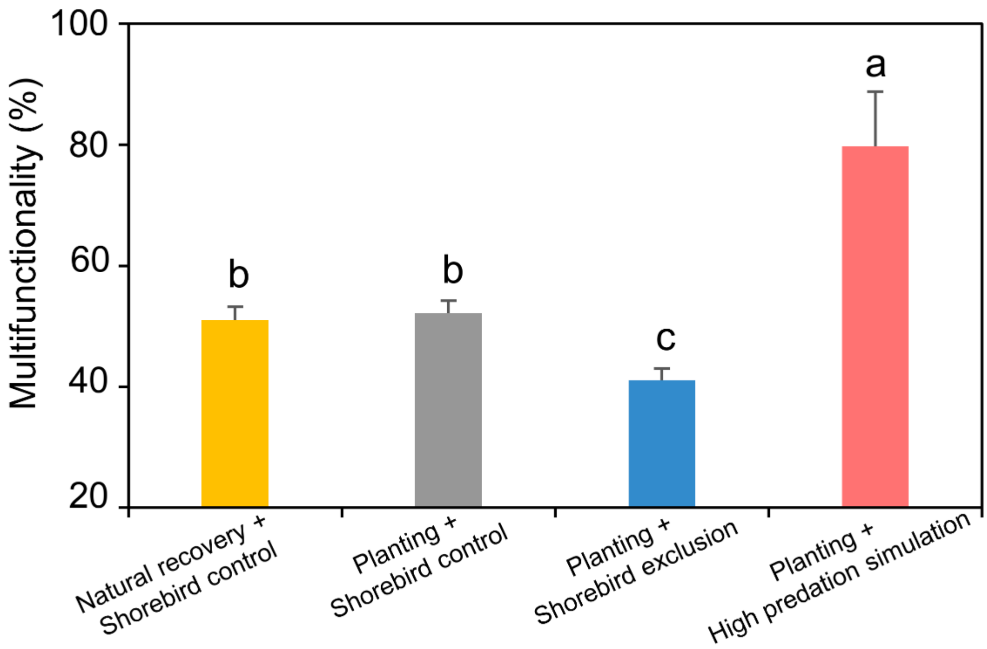


**Supplementary Fig. 18: Wetland multifunctionality in different restoration treatments.** Here, species richness of macrofauna and microbial OTUs, instead of macrofaunal biomass and microbial production, were used to calculate the average standardized multifunctionality index. Data are shown as means with error bars for standard errors ($n$ = 8 independent plots; one-way ANOVA; df = 3, 28, $F$ = 45.78, $P < 0.0001$). Bars sharing a letter are not significantly different from one another based on Tukey's HSD multiple comparisons ($P < 0.05$; detailed statistical results are provided in Supplementary Table 21).

**Supplementary Table 1: Shorebird and non-shorebird species observed at the restoration site from 2019 to 2021**

| Bird group | Foraging pattern | Species observed |
|---|---|---|
| Shorebird | Crab predators | *Numenius phaeopus, Calidris tenuirostris, Xenus cinereus, Tringa nebularia, Numenius arquata, Tringa stagnatilis, Tringa brevipes, Numenius minutus, Limosa limosa, Tringa erythropus, Calidris ferruginea, Limnodromus scolopaceus* |
| | Non-crab predators | *Charadrius alexandrinus, Calidris alpina, Calidris ruficollis, Eurynorhynchus pygmeus, Calidris acuminata, Calidris alba, Limicola falcinellus, Arenaria interpres, Actitis hypoleucos, Calidris minuta, Tringa tetanus, Charadrius dubius, Charadrius mongolus, Charadrius leschenaultii, Pluvialis fulva, Pluvialis squatarola, Charadrius placidus* |
| Non-shorebird | Crab predators | *Ardea cinerea, Ardeidae* spp., *Platalea leucorodia, Nycticorax nycticorax, Larus argentatus, Chlidonias leucopterus, Larus canus, Larus saundersi, Sterna hirundo, Chlidonias hybrida, Larus crassirostris, Larus ridibundus* |
| | Non-crab predators | *Hirundo rustica, Pica pica, Elanus caeruleus, Falco peregrinus, Pandion haliaetus, Anas platyrhynchos, Fringilla montifringilla* |

**Supplementary Table 2: The 12 ecosystem functions considered in this study and their corresponding ecosystem services and proxies measured in this study**

| Ecosystem function | Ecosystem service | Proxy measured | References |
|---|---|---|---|
| Primary production | Provisioning: fiber and biomass fuel | Plant biomass | Barbier *et al.* 2011, Michaletz *et al.* 2014, Ramus *et al.* 2017 |
| Secondary production | Provisioning: food; Supporting: fisheries | Macrofaunal biomass | Barbier *et al.* 2011, Ramus *et al.* 2017 |
| Microbial production | Supporting: nutrient cycling | Microbial biomass | Zhang et al. 2019, Garland *et al.* 2021 |
| Wave dissipation | Regulating: storm protection | Rate of gypsum block dissolution | Barbier *et al.* 2011, Ramus *et al.* 2017 |
| Marsh infiltration | Regulating: water runoff | Infiltration rate of water through marsh soils | Hemond *et al.* 1984, Hensel *et al.* 2013 |
| Sediment accretion | Regulating: erosion | Sediment accretion rate | Barbier *et al.* 2011, Ramus *et al.* 2017, Thomsen *et al.* 2019 |
| Soil respiration | Regulating: soil health | Rate of soil $CO_2$ release | Bastida *et al.* 2016, Lozano *et al.* 2021 |
| Nitrogen mineralization | Regulating: soil fertility | Changes in soil inorganic nitrogen per day | Schimel J.P. and Bennett J. 2004, Risch *et al.* 2019 |
| Litter decomposition | Supporting: nutrient cycling | Rate of plant litter biomass change | Barbier et al. 2011, Hensel *et al.* 2013, Ramus *et al.* 2017 |

| | | | |
|---|---|---|---|
| Sediment carbon burial | Regulating: climate change | Increases in the organic carbon of surface accreted sediment per unit area per month | Schlesinger 1999, Rattan 2008, Christianen *et al.* 2022 |
| Nitrogen accumulation | Supporting: waste treatment | Increases in sediment soil total nitrogen per unit area per month | Craft et al. 2009, Zhang *et al.* 2019, Geurts *et al.* 2020 |
| Soil heavy metal reduction | Supporting: soil pollution mitigation | Changes in the Nemerow multifactor pollution index | Abdullah *et al.* 1972, Zhang *et al.* 2021, Yan *et al.* 2022 |

**Supplementary Table 3: Estimated parameters of the meta-regression models for the effect size of birds on crab abundance against the abundances of different groups of birds, as well as bird species richness.**

| **Bird taxa** | **Intercept** | | | **Slope** | | |
|---|---|---|---|---|---|---|
| | **Estimate** | **SE** | ***P*-value** | **Estimate** | **SE** | ***P*-value** |
| **Shorebirds** | -0.6649 | 0.1310 | < 0.0001 | -0.0006 | 0.0002 | *P* < 0.0001 |
| Crab predators | -0.7492 | 0.1235 | < 0.0001 | -0.0011 | 0.0003 | *P* < 0.0001 |
| Non-crab predators | -0.7721 | 0.1342 | < 0.0001 | -0.0012 | 0.0003 | 0.0003 |
| **Non-shorebirds** | -1.1321 | 0.1153 | < 0.0001 | 0.0007 | 0.0007 | 0.3014 |
| Crab predators | -1.1050 | 0.1122 | < 0.0001 | 0.0005 | 0.0007 | 0.5136 |
| Non-crab predators | -1.1406 | 0.1063 | < 0.0001 | 0.0047 | 0.0029 | 0.6995 |
| *Charadrius alexandrinus* | -0.7180 | 0.1259 | < 0.0001 | -0.0012 | 0.0003 | 0.0001 |
| *Calidris alpina* | -0.8846 | 0.1235 | < 0.0001 | -0.0017 | 0.0007 | 0.0167 |
| *Calidris tenuirostris* | -0.8155 | 0.1195 | < 0.0001 | -0.0029 | 0.0009 | 0.0012 |
| *Numenius phaeopus* | -0.7617 | 0.1242 | < 0.0001 | -0.0044 | 0.0013 | 0.0004 |
| *Ardeidae* spp. | -1.1021 | 0.1107 | < 0.0001 | 0.0005 | 0.0008 | 0.5181 |
| *Xenus cinereus* | -0.8489 | 0.1392 | < 0.0001 | -0.0054 | 0.0024 | 0.0251 |
| *Calidris ruficollis* | -1.0318 | 0.1065 | < 0.0001 | -0.0020 | 0.0017 | 0.2160 |
| *Hirundo rustica* | -1.1344 | 0.1055 | < 0.0001 | 0.0046 | 0.0029 | 0.1077 |
| Species richness | -1.3932 | 0.2362 | < 0.0001 | 0.0355 | 0.0239 | 0.1376 |

**Supplementary Table 4: Summary statistics of one-way ANOVAs on the effects of different experimental treatments on crab abundance in the shorebirds' non-consumptive effects simulation experiment.**

| Sampling date | df | *F*-ratio | *P*-value |
|---|---|---|---|
| 18-Jun-23 | 2,  21 | 0.263 | 0.7713 |
| 20-Jun-23 | 2, 21 | 0.156 | 0.8562 |
| 27-Jun-23 | 2, 21 | 25.14 | < 0.0001 |
| 4-Jul-23 | 2, 21 | 49.921 | < 0.0001 |
| 10-Jul-23 | 2, 21 | 63.964 | < 0.0001 |

**Supplementary Table 5: Summary statistics of multiple comparisons on the effects of different experimental treatments on crab abundance in the shorebirds' non-consumptive effects simulation experiment.**

| Sampling date | Pairwise comparison | Diff | Lwr | Upr | *P*-value |
|---|---|---|---|---|---|
| 18-Jun-23 | PC *vs.* C | 0.625 | -1.6739 | 2.9239 | 0.7745 |
| | SM *vs.* C | 0.125 | -2.1739 | 2.4239 | 0.9897 |
| | SM *vs.* PC | -0.500 | -2.7989 | 1.7989 | 0.8484 |
| 20-Jun-23 | PC *vs.* C | 0.375 | -1.970 | 2.7207 | 0.9147 |
| | SM *vs.* C | -0.125 | -2.470 | 2.2207 | 0.9901 |
| | SM *vs.* PC | -0.500 | -2.8457 | 1.8457 | 0.8539 |
| 27-Jun-23 | PC *vs.* C | -1.250 | -2.9508 | 0.4508 | 0.1774 |
| | SM *vs.* C | -4.625 | -6.3258 | -2.9241 | < 0.0001 |
| | SM *vs.* PC | -3.375 | -5.0758 | -1.6741 | 0.0001 |
| 4-Jul-23 | PC *vs.* C | -1.375 | -2.9608 | 0.2108 | 0.0970 |
| | SM *vs.* C | -6.000 | -7.5858 | -4.4141 | < 0.0001 |
| | SM *vs.* PC | -4.625 | -6.2108 | -3.0391 | < 0.0001 |
| 10-Jul-23 | PC *vs.* C | -1.000 | -2.5281 | 0.5281 | 0.2478 |
| | SM *vs.* C | -6.375 | -7.9031 | -4.8468 | < 0.0001 |
| | SM *vs.* PC | -5.375 | -6.9031 | -3.8468 | < 0.0001 |

Note: C, PC, and SM refers to control, procedural control, and shorebird model treatments, respectively. Diff refers to the difference in mean value between two types of treatment. Lwr and Upr refer to the lower and upper bounds of 95% confidence interval, respectively.

**Supplementary Table 6: Summary statistics of multiple comparisons on the effects of different restoration treatments on the rate of plant abundance change.**

| **Pairwise comparison** | **Diff** | **Lwr** | **Upr** | ***P*-value** |
|---|---|---|---|---|
| PL + BE *vs.* PL | -3.4406 | -4.8689 | -2.0123 | < 0.0001 |
| PL + HP *vs.* PL | 2.7437 | 1.3154 | 4.1720 | 0.0002 |
| PL + HP *vs.* PL +BE | 6.1843 | 4.7560 | 7.6126 | < 0.0001 |

Note: PL refers to planting + shorebird control treatments; NR refers to natural recovery + shorebird control treatments; PL + BE refers to planting + shorebird exclusion treatments; and PL + HP refers to planting + high predation simulation treatments. Diff refers to the difference in mean value between two types of treatment. Lwr and Upr refer to the lower and upper bounds of 95% confidence interval, respectively.

**Supplementary Table 7: Summary statistics of one-way ANOVAs (or Wilcoxon tests) on the effects of different restoration treatments on wetland functions.**

| **Wetland functions** | **df** | ***F*-ratio (V)** | ***P*-value** |
|---|---|---|---|
| Primary production | - | 36 | 0.01427 |
| Secondary production | - | 528 | < 0.0001 |
| Microbial production | 3, 28 | 1.822 | 0.166 |
| Wave dissipation | 3, 28 | 6.111 | 0.0025 |
| Marsh infiltration | 3, 28 | 3.523 | 0.0277 |
| Sediment accretion. | 3, 28 | 68.46 | < 0.0001 |
| Soil respiration | 3, 28 | 168.3 | < 0.0001 |
| Nitrogen mineralization | 3, 28 | 9.651 | 0.000154 |
| Litter decomposition | 3, 28 | 13.23 | < 0.0001 |
| Sediment carbon burial | 3, 28 | 37.67 | < 0.0001 |
| Nitrogen accumulation | 3, 28 | 53.57 | < 0.0001 |
| Heavy metal reduction | 3, 28 | 28.79 | < 0.0001 |

Note: "V" stands for the Wilcoxon rank sum statistic (for primary production and secondary production).

**Supplementary Table 8: Summary statistics of multiple comparisons on the effects of different restoration treatments on wetland functions.**

| **Wetland function** | **Pairwise comparison** | **Diff** | **Lwr** | **Upr** | ***P*-value** |
|---|---|---|---|---|---|
| Primary production | PL *vs.* NR | - | - | - | - |
| | PL + BE *vs.* NR | - | - | - | - |
| | PL + HP *vs.* NR | - | - | - | 0.0012 |
| | PL + BE *vs.* PL | - | - | - | - |
| | PL + HP *vs.* PL | - | - | - | 0.0012 |
| | PL + HP *vs.* PL + BE | - | - | - | 0.0012 |
| Secondary production | PL *vs.* NR | - | - | - | 1.0000 |
| | PL + BE *vs.* NR | - | - | - | 1.0000 |
| | PL + HP *vs.* NR | - | - | - | 0.0037 |
| | PL + BE *vs.* PL | - | - | - | 1.0000 |
| | PL + HP *vs.* PL | - | - | - | 0.0321 |
| | PL + HP *vs.* PL + BE | - | - | - | 0.0019 |
| Microbial production | PL *vs.* NR | 0.1840 | -4.7686 | 5.1366 | 0.9996 |
| | PL + BE *vs.* NR | -3.2368 | -8.1895 | 1.7157 | 0.3017 |
| | PL + HP *vs.* NR | 0.4376 | 0.2207 | 5.3902 | 0.9949 |
| | PL + BE *vs.* PL | -3.4208 | -4.5150 | 1.5317 | 0.2568 |
| | PL + HP *vs.* PL | 0.2536 | -8.3735 | 5.2062 | 0.9990 |
| | PL + HP *vs.* PL + BE | 3.6745 | -4.6990 | 8.6271 | 0.2028 |
| Wave dissipation | PL *vs.* NR | 0.2062 | -0.7807 | 1.1932 | 0.9400 |
| | PL + BE *vs.* NR | -0.2246 | -1.2116 | 0.7620 | 0.9243 |
| | PL + HP *vs.* NR | 1.2077 | 0.2207 | 2.1947 | 0.0120 |
| | PL + BE *vs.* PL | -0.4308 | -1.4178 | 0.5561 | 0.6369 |
| | PL + HP *vs.* PL | 1.0015 | 0.0144 | 1.9885 | 0.0457 |

| | | | | | |
|---|---|---|---|---|---|
| | PL + HP *vs.* PL + BE | 1.4323 | 0.4453 | 2.4194 | 0.0024 |
| Marsh infiltration | PL *vs.* NR | 0.0018 | -0.4610 | 0.4648 | 0.999 |
| | PL + BE *vs.* NR | 0.0438 | -0.4190 | 0.5068 | 0.0731 |
| | PL + HP *vs.* NR | -0.4330 | -0.8959 | 0.0299 | 0.0731 |
| | PL + BE *vs.* PL | 0.0420 | -0.4209 | 0.5049 | 0.9945 |
| | PL + HP *vs.* PL | -0.4348 | -0.8978 | 0.0280 | 0.0714 |
| | PL + HP *vs.* PL + BE | -0.4768 | -0.9398 | -0.0139 | 0.0416 |
| Sediment accretion | PL *vs.* NR | 0.0011 | -0.0241 | 0.0263 | 0.9993 |
| | PL + BE *vs.* NR | -0.0440 | -0.0692 | -0.0187 | 0.0003 |
| | PL + HP *vs.* NR | 0.0855 | 0.0602 | 0.1107 | < 0.0001 |
| | PL + BE *vs.* PL | -0.0451 | -0.0703 | -0.0198 | 0.00021 |
| | PL + HP *vs.* PL | 0.0843 | 0.05910 | 0.1096 | < 0.0001 |
| | PL + HP *vs.* PL + BE | 0.1295 | 0.1042 | 0.1547 | < 0.0001 |
| Soil respiration | PL *vs.* NR | 0.0235 | -0.0575 | 0.1045 | 0.8578 |
| | PL + BE *vs.* NR | 0.2837 | 0.2026 | 0.3648 | < 0.0001 |
| | PL + HP *vs.* NR | 0.5828 | 0.5017 | 0.6639 | < 0.0001 |
| | PL + BE *vs.* PL | 0.2602 | 0.1791 | 0.3413 | < 0.0001 |
| | PL + HP *vs.* PL | 0.5593 | 0.4782 | 0.6404 | < 0.0001 |
| | PL + HP *vs.* PL + BE | 0.2991 | 0.2180 | 0.3802 | < 0.0001 |
| Nitrogen mineralization | PL *vs.* NR | -0.0045 | -0.0512 | 0.0420 | 0.9931 |
| | PL + BE *vs.* NR | -0.0335 | -0.0801 | 0.0131 | 0.2266 |
| | PL + HP *vs.* NR | 0.0562 | 0.0096 | 0.1028 | 0.0135 |
| | PL + BE *vs.* PL | -0.0289 | -0.0755 | 0.01770 | 0.3456 |
| | PL + HP *vs.* PL | 0.0608 | 0.0141 | 0.1074 | 0.0069 |
| | PL + HP *vs.* PL + BE | 0.0897 | 0.0431 | 0.1363 | < 0.0001 |
| Litter decomposition | PL *vs.* NR | -0.0025 | -0.0226 | 0.0176 | 0.9863 |
| | PL + BE *vs.* NR | -0.0148 | -0.0350 | 0.0052 | 0.2065 |

| | | | | | |
|---|---|---|---|---|---|
| | PL + HP *vs.* NR | 0.0298 | 0.0097 | 0.0500 | 0.0019 |
| | PL + BE *vs.* PL | -0.0123 | -0.0325 | 0.0077 | 0.3540 |
| | PL + HP *vs.* PL | 0.0323 | 0.0122 | 0.0525 | 0.0008 |
| | PL + HP *vs.* PL + BE | 0.0447 | 0.0245 | 0.0649 | < 0.0001 |
| Sediment carbon burial | PL *vs.* NR | -0.3838 | -13.732 | 12.9651 | 0.9998 |
| | PL + BE *vs.* NR | -19.2300 | -32.579 | -5.8809 | 0.0026 |
| | PL + HP *vs.* NR | 31.9153 | 18.5663 | 45.2644 | < 0.0001 |
| | PL + BE *vs.* PL | -18.8461 | -32.195 | -5.4971 | 0.0032 |
| | PL + HP *vs.* PL | 32.2992 | 18.9502 | 45.6482 | < 0.0001 |
| | PL + HP *vs.* PL + BE | 51.1453 | 37.7963 | 64.4944 | < 0.0001 |
| Nitrogen accumulation | PL *vs.* NR | 0.3633 | -1.0882 | 1.8149 | 0.9025 |
| | PL + BE *vs.* NR | -2.0335 | -3.4851 | -0.5818 | 0.0035 |
| | PL + HP *vs.* NR | 4.5260 | 3.0743 | 5.9776 | < 0.0001 |
| | PL + BE *vs.* PL | -2.3968 | -3.8484 | -0.9452 | 0.0005 |
| | PL + HP *vs.* PL | 4.1626 | 2.7110 | 5.6142 | < 0.0001 |
| | PL + HP *vs.* PL + BE | 6.5595 | 5.1078 | 8.0111 | < 0.0001 |
| Heavy metal reduction | PL *vs.* NR | -0.0153 | -0.1148 | 0.0841 | 0.9742 |
| | PL + BE *vs.* NR | -0.2021 | -0.0350 | -0.1026 | < 0.0001 |
| | PL + HP *vs.* NR | 0.1342 | -0.3016 | 0.2337 | 0.0050 |
| | PL + BE *vs.* PL | -0.1867 | 0.0347 | -0.0872 | 0.0001 |
| | PL + HP *vs.* PL | 0.1496 | 0.0501 | 0.2491 | 0.0017 |
| | PL + HP *vs.* PL + BE | 0.3363 | 0.2368 | 0.4358 | < 0.0001 |

Note: PL refers to planting + shorebird control treatments; NR refers to natural recovery + shorebird control treatments; PL + BE refers to planting + shorebird exclusion treatments; and PL + HP refers to planting + high predation simulation treatments. Diff refers to the difference in mean value between two types of treatment. Lwr and Upr refer to the lower and upper bounds of

95% confidence interval, respectively. -, not available for multiple comparisons following a nonparametric Wilcoxon test (for Diff, Upr, and Lwr) and for *P* values when values in both groups of treatments are all zeros.

**Supplementary Table 9: Summary statistics of the linear or quadratic regression models for ecosystem functions against crab abundance.**

| Ecosystem functions | df | *F*-statistic | *R*-squared | *P*-value |
|---|---|---|---|---|
| Secondary production | 1, 22 | 0.3872 | 0.0173 | 0.5402 |
| Microbial production | 1, 22 | 17.75 | 0.4465 | 0.0003 |
| Wave dissipation | 1, 22 | 1.899 | 0.07946 | 0.182 |
| Marsh infiltration | 1, 22 | 1.976 | 0.1584 | 0.1636 |
| Sediment accretion | 1, 22 | 114.70 | 0.8391 | < 0.0001 |
| Soil respiration | 1, 22 | 53.19 | 0.7074 | < 0.0001 |
| Nitrogen mineralization | 1, 22 | 4.882 | 0.1816 | 0.05485 |
| Litter decomposition | 1, 22 | 6.169 | 0.219 | 0.0211 |
| Sediment carbon burial | 1, 22 | 157.90 | 0.8777 | < 0.0001 |
| Nitrogen accumulation | 1, 22 | 73.80 | 0.7703 | < 0.0001 |
| Heavy metal reduction | 1, 22 | 30.40 | 0.5802 | < 0.0001 |
| Multifunctionality | 1, 22 | 29.58 | 0.738 | < 0.0001 |

**Supplementary Table 10: Summary statistics of the linear or quadratic regression models for ecosystem functions against primary production.**

| Ecosystem functions | df | *F*-statistic | *R*-squared | *P*-value |
|---|---|---|---|---|
| Secondary production | 1, 30 | 19.45 | 0.5729 | < 0.0001 |
| Microbial production | 1, 30 | 0.669 | 0.02181 | 0.4199 |
| Wave dissipation | 1, 30 | 33.22 | 0.5255 | < 0.0001 |
| Marsh infiltration | 1, 30 | 15.12 | 0.3351 | 0.0005 |
| Sediment accretion | 1, 30 | 119.7 | 0.7997 | < 0.0001 |
| Soil respiration | 1, 30 | 36.89 | 0.7179 | < 0.0001 |
| Nitrogen mineralization | 1, 30 | 42.25 | 0.5848 | < 0.0001 |
| Litter decomposition | 1, 30 | 33.28 | 0.5259 | < 0.0001 |
| Sediment carbon burial | 1, 30 | 109.9 | 0.7855 | < 0.0001 |
| Nitrogen accumulation | 1, 30 | 108.2 | 0.7829 | < 0.0001 |
| Heavy metal reduction | 1, 30 | 28.65 | 0.4885 | < 0.0001 |
| Multifunctionality | 1, 30 | 122 | 0.8938 | < 0.0001 |

**Supplementary Table 11: Summary statistics of multiple comparisons on the effects of different restoration treatments on the averaged multifunctionality index.**

| **Pairwise comparison** | **Diff** | **Lwr** | **Upr** | ***P*-value** |
|---|---|---|---|---|
| PL *vs.* NR | 0.0113 | -0.0781 | 0.1008 | 0.9853 |
| PL + BE *vs.* NR | -0.0869 | -0.1764 | 0.0025 | 0.0491 |
| PL + HP *vs.* NR | 0.3048 | 0.2153 | 0.3943 | < 0.0001 |
| PL + BE *vs.* PL | -0.0983 | -0.1878 | -0.0087 | 0.0272 |
| PL + HP *vs.* PL | 0.2934 | 0.2039 | 0.3830 | 0.0017 |
| PL + HP *vs.* PL + BE | 0.3917 | 0.3022 | 0.4813 | < 0.0001 |

Note: Abbreviations follow Supplementary Table 8.

**Supplementary Table 12: Summary statistics of one-way ANOVAs on the effects of different restoration treatments on the effective number of functions.**

| **Order** | **df** | ***F*-ratio** | ***P*-value** |
|---|---|---|---|
| $q = 1$ | 3, 28 | 42.46 | < 0.0001 |
| $q = 2$ | 3, 28 | 43.58 | < 0.0001 |
| $q = 3$ | 3, 28 | 49.80 | < 0.0001 |
| $q = 4$ | 3, 28 | 47.22 | < 0.0001 |
| $q = 5$ | 3, 28 | 47.90 | < 0.0001 |

**Supplementary Table 13: Summary statistics of multiple comparisons on the effects of different restoration treatments on the effective number of functions.**

| Order | Pairwise comparison | Diff | Lwr | Upr | *P*-value |
|---|---|---|---|---|---|
| *q* = 1 | PL *vs.* NR | 0.0645 | -0.55458 | 0.6836 | 0.9917 |
| | PL + BE *vs.* NR | -0.9082 | -1.5273 | -0.2891 | 0.0022 |
| | PL + HP *vs.* NR | 1.6101 | 0.9910 | 2.2293 | < 0.0001 |
| | PL + BE *vs.* PL | -0.9728 | -1.59194 | -0.3536 | 0.0010 |
| | PL + HP *vs.* PL | 1.5456 | 0.9264 | 2.1647 | < 0.0001 |
| | PL + HP *vs.* PL + BE | 2.5184 | 1.8993 | 3.1375 | < 0.0001 |
| *q* = 2 | PL *vs.* NR | 0.1404 | -0.636 | 0.9177 | 0.9598 |
| | PL + BE *vs.* NR | -1.2574 | -2.0346 | -0.4802 | 0.0007 |
| | PL + HP *vs.* NR | 1.9693 | 1.1920 | 2.7465 | < 0.0001 |
| | PL + BE *vs.* PL | -1.3979 | -2.1751 | -0.6206 | 0.0002 |
| | PL + HP *vs.* PL | 1.8288 | 1.0516 | 2.6060 | < 0.0001 |
| | PL + HP *vs.* PL + BE | 3.2267 | 2.4495 | 4.0039 | < 0.0001 |
| *q* = 3 | PL *vs.* NR | 0.1541 | -0.6392 | 0.9474 | 0.9509 |
| | PL + BE *vs.* NR | -1.3813 | -2.1746 | -0.5880 | 0.0003 |
| | PL + HP *vs.* NR | 2.1408 | 1.3475 | 2.9342 | < 0.0001 |
| | PL + BE *vs.* PL | -1.5354 | -2.3288 | -0.7421 | < 0.0001 |
| | PL + HP *vs.* PL | 1.9867 | 1.1934 | 2.7800 | < 0.0001 |
| | PL + HP *vs.* PL + BE | 3.5222 | 2.7289 | 4.3155 | < 0.0001 |
| *q* = 4 | PL *vs.* NR | 0.4603 | -1.878 | 2.7989 | 0.9491 |
| | PL + BE *vs.* NR | -3.6773 | -6.0158 | -1.3387 | 0.0010 |
| | PL + HP *vs.* NR | 6.3821 | 4.0436 | 8.7206 | < 0.0001 |
| | PL + BE *vs.* PL | -4.1377 | -6.4762 | -1.7991 | 0.0002 |
| | PL + HP *vs.* PL | 5.9217 | 3.5832 | 8.26029 | < 0.0001 |

| | | | | | |
|---|---|---|---|---|---|
| | PL + HP *vs.* PL + BE | 10.0594 | 7.7209 | 12.3980 | < 0.0001 |
| *q* = 5 | PL *vs.* NR | 0.1766 | -0.6820 | 1.0353 | 0.9425 |
| | PL + BE *vs.* NR | -1.4301 | -2.2888 | -0.5714 | 0.0005 |
| | PL + HP *vs.* NR | 2.3033 | 1.4447 | 3.1620 | < 0.0001 |
| | PL + BE *vs.* PL | -1.6068 | -2.4654 | -0.7481 | 0.0001 |
| | PL + HP *vs.* PL | 2.1267 | 1.2680 | 2.9853 | < 0.0001 |
| | PL + HP *vs.* PL + BE | 3.7335 | 2.8748 | 4.5921 | < 0.0001 |

Note: Abbreviations follow Supplementary Table 8.

**Supplementary Table 14: Summary statistics of one-way ANOVAs on the effects of different restoration treatments on the effective multifunctionality.**

| Order | df | *F*-ratio | *P*-value |
|---|---|---|---|
| $q = 0$ | 3, 28 | 54.25 | < 0.0001 |
| $q = 1$ | 3, 28 | 59.35 | < 0.0001 |
| $q = 2$ | 3, 28 | 59.12 | < 0.0001 |
| $q = 3$ | 3, 28 | 59.70 | < 0.0001 |
| $q = 4$ | 3, 28 | 55.22 | < 0.0001 |
| $q = 5$ | 3, 28 | 57.41 | < 0.0001 |

**Supplementary Table 15: Summary statistics of multiple comparisons on the effects of different restoration treatments on the effective multifunctionality.**

| Order | Pairwise comparison | Diff | Lwr | Upr | ***P*-value** |
|---|---|---|---|---|---|
| *q* = 0 | PL *vs.* NR | 0.0113 | -0.0781 | 0.1008 | 0.9853 |
| | PL + BE *vs.* NR | -0.0869 | -0.1764 | 0.0025 | 0.0593 |
| | PL + HP *vs.* NR | -0.0869 | 0.2153 | 0.3943 | < 0.0001 |
| | PL + BE *vs.* PL | 0.3048 | -0.1878 | -0.0087 | 0.02725 |
| | PL + HP *vs.* PL | -0.0983 | 0.2039 | 0.3830 | < 0.0001 |
| | PL + HP *vs.* PL + BE | 0.3917 | 0.3022 | 0.4813 | < 0.0001 |
| *q* = 1 | PL *vs.* NR | 0.1370 | -1.043 | 1.3175 | < 0.0001 |
| | PL + BE *vs.* NR | -1.1855 | -2.366 | -0.0049 | 0.9887 |
| | PL + HP *vs.* NR | 4.2090 | 3.028 | 5.3896 | 0.0487 |
| | PL + BE *vs.* PL | -1.3225 | -2.5030 | -0.1420 | < 0.0001 |
| | PL + HP *vs.* PL | 4.0720 | 2.8915 | 5.2525 | 0.0236 |
| | PL + HP *vs.* PL + BE | 5.3945 | 4.2140 | 6.5751 | < 0.0001 |
| *q* = 2 | PL *vs.* NR | 0.1623 | -1.0406 | 1.3652 | 0.9825 |
| | PL + BE *vs.* NR | -1.2554 | -2.4583 | -0.0525 | 0.0383 |
| | PL + HP *vs.* NR | 4.2557 | 3.0527 | 5.4586 | < 0.0001 |
| | PL + BE *vs.* PL | -1.4177 | -2.6206 | -0.2148 | 0.0161 |
| | PL + HP *vs.* PL | 4.0934 | 2.8904 | 5.2963 | < 0.0001 |
| | PL + HP *vs.* PL + BE | 5.5111 | 4.3082 | 6.7141 | < 0.0001 |
| *q* = 3 | PL *vs.* NR | 0.1635 | -1.0336 | 1.3608 | 0.9819 |
| | PL + BE *vs.* NR | -1.2663 | -2.4636 | -0.0691 | 0.0351 |
| | PL + HP *vs.* NR | 4.2501 | 3.0529 | 5.4474 | < 0.0001 |
| | PL + BE *vs.* PL | -1.4299 | -2.6271 | -0.2326 | 0.0145 |
| | PL + HP *vs.* PL | 4.0866 | 2.8893 | 5.2838 | < 0.0001 |

| | | | | | |
|---|---|---|---|---|---|
| | PL + HP *vs.* PL + BE | 5.5165 | 4.3193 | 6.7137 | < 0.0001 |
| *q* = 4 | PL *vs.* NR | 0.3817 | -2.4642 | 3.2277 | 0.9828 |
| | PL + BE *vs.* NR | -2.7916 | -5.6376 | 0.0544 | 0.0560 |
| | PL + HP *vs.* NR | -2.7916 | 6.9338 | 12.6258 | < 0.0001 |
| | PL + BE *vs.* PL | 9.7798 | -6.019 | -0.3273 | 0.0244 |
| | PL + HP *vs.* PL | -3.1733 | 6.5520 | 12.2440 | < 0.0001 |
| | PL + HP *vs.* PL + BE | 12.5714 | 9.7254 | 15.4174 | < 0.0001 |
| *q* = 5 | PL *vs.* NR | 0.16742 | -1.0371 | 1.3720 | 0.9810 |
| | PL + BE *vs.* NR | -1.2392 | -2.4438 | -0.0346 | 0.0419 |
| | PL + HP *vs.* NR | 4.2007 | 2.9962 | 5.4053 | < 0.0001 |
| | PL + BE *vs.* PL | -1.4066 | -2.6112 | -0.2020 | 0.0173 |
| | PL + HP *vs.* PL | 4.0333 | 2.8287 | 5.2379 | < 0.0001 |
| | PL + HP *vs.* PL + BE | 5.4400 | 4.2354 | 6.6446 | < 0.0001 |

Note: Abbreviations follow Supplementary Table 8.

**Supplementary Table 16: Summary statistics of multiple comparisons on the effects of different restoration treatments on above- and belowground multifunctionality.**

| Multifunctionality | Pairwise comparison | Diff | Lwr | Upr | *P*-value |
|---|---|---|---|---|---|
| Aboveground multifunctionality | PL *vs.* NR | 0.0156 | -0.1248 | 0.1562 | 0.9899 |
| | PL + BE *vs.* NR | -0.0654 | -0.2059 | 0.07511 | 0.5884 |
| | PL + HP *vs.* NR | 0.3604 | 0.21989 | 0.5009 | < 0.0001 |
| | PL + BE *vs.* PL | -0.0810 | -0.2216 | 0.0594 | 0.4082 |
| | PL + HP *vs.* PL | 0.3447 | 0.20420 | 0.4852 | < 0.0001 |
| | PL + HP *vs.* PL + BE | 0.4258 | 0.2853 | 0.5663 | < 0.0001 |
| Belowground multifunctionality | PL *vs.* NR | 0.0081 | -0.0842 | 0.1006 | 0.9949 |
| | PL + BE *vs.* NR | -0.0868 | -0.1792 | 0.0055 | 0.0471 |
| | PL + HP *vs.* NR | 0.3198 | 0.2274 | 0.4122 | < 0.0001 |
| | PL + BE *vs.* PL | -0.0950 | -0.1874 | -0.0026 | 0.0421 |
| | PL + HP *vs.* PL | 0.3116 | 0.2192 | 0.40411 | < 0.0001 |
| | PL + HP *vs.* PL + BE | 0.4067 | 0.3143 | 0.4991 | < 0.0001 |

Note: Abbreviations follow Supplementary Table 8.

**Supplementary Table 17: List of the papers included in the meta-analysis on the effects of crab grazers on native plant biomass.**

| No. | Year | Title | Authors | Journal | Volume | Page/number |
|---|---|---|---|---|---|---|
| 01 | 2021 | Interactive effects of crab herbivory and spring drought on a *Phragmites australis*-dominated salt marsh in the Yellow River Delta | Zhang *et al.* | Science of the Total Environment | 766 | 144254 |
| 02 | 2020 | Hydrological connectivity and herbivores control the autochthonous producers of coastal salt marshes | Yin *et al.* | Marine Pollution Bulletin | 160 | 111638 |
| 03 | 2019 | Macrophytes and crabs affect nitrogen transformations in salt marshes of the Yangtze River Estuary | Zhang *et al.* | Estuarine, Coastal and Shelf Science | 225 | 106242 |
| 04 | 2022 | Native herbivores indirectly facilitate the growth of | Xu *et al.* | Ecology | 103 | e3610 |

| | | | | | | |
|---|---|---|---|---|---|---|
| | | invasive Spartina in a eutrophic saltmarsh | | | | |
| 05 | 2019 | Weather fluctuations affect the impact of consumers on vegetation recovery following a catastrophic die-off | He *et al.* | Ecology | 100 | e02559 |
| 06 | 2017 | Natural enemies govern ecosystem resilience in the face of extreme droughts | He *et al.* | Ecology Letters | 20 | 194-201 |
| 07 | 2015 | Herbivory drives zonation of stress-tolerant marsh plants | He *et al.* | Ecology | 96 | 1318-1328 |

**Supplementary Table 18: Quantitative PCR primer information and reaction conditions***

| Primer name | Primer sequence (5' - 3') | | Thermal profile | Product fragment size |
|---|---|---|---|---|
| *Eub338_Eub518* | F1norA | ACTCCTACGGGAGGCAGCAG | 95°C/5 min; 35 cycles of 95°C/30 s, 60°C/30 s, 72°C/1 min | 191 bp |
| | R2norA | ATTACCGCGGCTGCTGG | | |

* All qPCR assays were run in triplicate. Total microbial genomic DNA was extracted using the FastDNA® Spin Kit for Soil (MP Biomedicals, Norcross, GA, USA) according to the manufacturer's instructions. The quality of extracted DNA was verified by 1.0 % agarose gel electrophoresis, and the DNA concentration was determined using a NanoDrop ND-2000 spectrophotometer (Thermo Fisher Scientific Inc., Waltham, MA, USA). The qPCR reaction system (a total of 20 μL) contained 10 μL of Master Mix (2 × ChamQ Universal SYBR qPCR Master Mix, Vazyme Biotech Co., Ltd., Nanjing, China), 0.8 μL of each primer (5 μmol $L^{-1}$), 1.0 μL of template DNA, and 7.4 μL of dd$H_2O$. Standard curves were constructed using 10-fold serially diluted standard plasmids amplified by qPCR in triplicate ($R^2$ = 0.996).

**Supplementary Table 19: Summary statistics of multiple comparisons on the effects of different restoration treatments on gypsum dissolution and the Nemerow multifactor pollution index.**

| **Multifunctionality** | **Pairwise comparison** | **Diff** | **Lwr** | **Upr** | ***P*-value** |
|---|---|---|---|---|---|
| Gypsum dissolution | PL *vs.* NR | -0.2062 | -1.1935 | 0.7810 | 0.9401 |
| | PL + BE *vs.* NR | 0.2237 | -0.7635 | 1.2110 | 0.9252 |
| | PL + HP *vs.* NR | -1.2075 | -2.1947 | -0.2202 | 0.0120 |
| | PL + BE *vs.* PL | 0.4300 | -0.5572 | 1.4172 | 0.6385 |
| | PL + HP *vs.* PL | -1.001 | -1.9885 | -0.0139 | 0.0459 |
| | PL + HP *vs.* PL + BE | -1.4312 | -2.4185 | -0.4439 | 0.0025 |
| Nemerow multifactor pollution index | PL *vs.* NR | 0.0154 | -0.0841 | 0.1149 | 0.9741 |
| | PL + BE *vs.* NR | 0.2021 | 0.1026 | 0.3017 | < 0.0001 |
| | PL + HP *vs.* NR | -0.1341 | -0.2337 | -0.0346 | 0.0051 |
| | PL + BE *vs.* PL | 0.1867 | 0.0872 | 0.2863 | 0.0001 |
| | PL + HP *vs.* PL | -0.14958 | -0.2491 | -0.0500 | 0.0017 |
| | PL + HP *vs.* PL + BE | -0.3363 | -0.4359 | -0.2368 | < 0.0001 |

Note: Abbreviations follow Supplementary Table 8.

**Supplementary Table 20: Summary statistics of multiple comparisons on the effects of different restoration treatments on macrofaunal species richness and microbial OTUs.**

| Wetland functions | Pairwise comparison | Diff | Lwr | Upr | *P*-value |
|---|---|---|---|---|---|
| Macrofaunal species richness | PL *vs.* NR | 0.875 | -2.0108 | 3.7608 | 0.8408 |
| | PL + BE *vs.* NR | 0.750 | -2.1358 | 3.6358 | 0.8924 |
| | PL + HP *vs.* NR | 2.000 | -0.8858 | 4.8858 | 0.2542 |
| | PL + BE *vs.* PL | -0.125 | -3.0108 | 2.7608 | 0.9993 |
| | PL + HP *vs.* PL | 1.125 | -1.7608 | 4.0108 | 0.7135 |
| | PL + HP *vs.* PL + BE | 1.250 | -1.6358 | 4.1358 | 0.6425 |
| Microbe OTUs | PL *vs.* NR | -505.625 | -8356.152 | 7344.902 | 0.9980 |
| | PL + BE *vs.* NR | 3295.875 | -4554.652 | 11146.402 | 0.6646 |
| | PL + HP *vs.* NR | 501.500 | -7349.027 | 8352.027 | 0.9980 |
| | PL + BE *vs.* PL | 3801.500 | -4049.027 | 11652.027 | 0.5570 |
| | PL + HP *vs.* PL | 1007.125 | -6843.402 | 8857.652 | 0.9849 |
| | PL + HP *vs.* PL + BE | -2794.375 | -10644.902 | 5056.152 | 0.7663 |

Note: Abbreviations follow Supplementary Table 8.

**Supplementary Table 21: Summary statistics of multiple comparisons on the effects of different restoration treatments on the averaged wetland multifunctionality when calculated using species richness of macrofauna and microbial OTUs, instead of macrofaunal biomass and microbial production.**

| **Pairwise comparison** | **Diff** | **Lwr** | **Upr** | ***P*-value** |
|---|---|---|---|---|
| PL *vs.* NR | 0.0112 | -0.0750 | 0.0973 | 0.9845 |
| PL + BE *vs.* NR | -0.0592 | -0.1454 | 0.0268 | 0.0459 |
| PL + HP *vs.* NR | 0.2795 | 0.1934 | 0.3657 | < 0.0001 |
| PL + BE *vs.* PL | -0.0704 | -0.1566 | 0.0157 | 0.0413 |
| PL + HP *vs.* PL | 0.2684 | 0.1822 | 0.3545 | < 0.0001 |
| PL + HP *vs.* PL + BE | 0.3388 | 0.2526 | 0.4249 | < 0.0001 |

Note: Abbreviations follow Supplementary Table 8.

## Appendix S1: Additional methods for quantifying microbial OTUs

### DNA extraction and PCR amplification

Total microbial genomic DNA was extracted from 32 soil samples using the FastDNA® Spin Kit for Soil (MP Biomedicals, Norcross, GA, U.S.) according to the manufacturer's instructions. The quality and concentration of DNA were determined by 1.0% agarose gel electrophoresis and a NanoDrop® ND-2000 spectrophotometer (Thermo Scientific Inc., USA) and kept at -80 ℃ prior to further use. The hypervariable region V3-V4 of the bacterial 16S rRNA gene were amplified with primer pairs 338F (5'-ACTCCTACGGGAGGCAGCAG-3') and 806R (5'-GGACTACHVGGGTWTCTAAT-3') (Liu *et al.* 2016) by an ABI GeneAmp® 9700 PCR thermocycler (ABI, CA, USA). The PCR reaction mixture (20 µL in total) included 4 µL of 5 × Fast Pfu buffer, 2 µL of 2.5 mM dNTPs, 0.8 µL of each primer (5 µM), 0.4 µL of FastPfu polymerase, 10 ng of template DNA, and 12.6 µL of $ddH_2O$. PCR amplification cycling conditions were as follows: initial denaturation at 95 ℃ for 3 minutes, followed by 32 cycles of denaturing at 95 ℃ for 30 s, annealing at 55 ℃ for 30 s, and extension at 72 ℃ for 45 s, and single extension at 72 ℃ for 10 min, and end at 4 ℃. All samples were amplified in triplicate. The PCR product was extracted from 2% agarose gel and purified using the AxyPrep DNA Gel Extraction Kit (Axygen Biosciences, Union City, CA, USA) according to the manufacturer's instructions and quantified using Quantus™ Fluorometer (Promega, USA).

### Sequencing and data processing

Purified amplicons were pooled in equimolar amounts and paired-end sequenced on an Illumina

MiSeq PE300 platform platform (Illumina, San Diego,USA) according to the standard protocols by Majorbio Bio-Pharm Technology Co. Ltd. (Shanghai, China). The raw 16S rRNA gene sequencing reads were demultiplexed, quality-filtered by fastp version 0.20.0, and merged by FLASH version 1.2.7 (Magoč *et al.* 2011) with the following criteria: (i) The reads (at any site receiving an average quality score of < 20) were truncated over a 50 bp sliding window (truncated reads shorter than 50 bp, as well as reads containing ambiguous characters, were discarded). (ii) Only overlapping sequences longer than 10 bp were assembled according to their overlapped sequence. The maximum mismatch ratio of the overlap region was 0.2. Reads that could not be assembled were discarded. (iii) Samples were distinguished according to the barcode and primers at the beginning and end of the sequences, with the sequence direction adjusted. The number of allowed mismatches per barcode and primer was 0 and 2, respectively.

Operational taxonomic units (OTUs) with 97% similarity cutoff (Stackebrandt *et al.* 1994, Edgar *et al.* 2013) were clustered using UPARSE version 7.1 (Edgar *et al.* 2013), and chimeric sequences were identified and removed. All optimized sequences were mapped to OTU representative sequences, and those with > 97% similarity to OTU representative sequences were selected to generate OTU table.

## Appendix S2: Additional methods for calculating the Nemerow multifactor pollution index

To calculate the Nemerow multifactor pollution index, we first calculated a pollution index for each pollutant using the following equation:

$$P_i = \frac{C_i}{C_s}$$

where $P_i$ is the index of pollutant $i$, $C_i$ is the concentration of pollutant $i$, and $C_s$ is the reference concentration value of pollutant $i$ (0.2, 15, 35, 100, 90, 35 and 40 mg $kg^{-1}$ for Cadmium, Arsenic, Lead, Zinc, Chromium, Copper, and Nickel, respectively, based on China National Soil Environmental Quality Standard). The Nemerow multifactor pollution index was then calculated as follows:

$$P = \sqrt{\frac{(P_i)^2_{max} - (\bar{P}_i)^2}{2}}$$

where $P$ is the Nemerow multifactor pollution index, $(P_i)_{max}$ is the maximum of $P_i$, and $\bar{P}_i$ is the mean value of $P_i$. A greater value of the Nemerow multifactor pollution index denotes a higher risk of pollution (He *et al.* 1998, Kowalska *et al.* 2016, Martínez-Guijarro *et al.* 2019).

## Supplementary References


Abdullah, M. I., Royle, L. G. & Morris A.W. Heavy metal concentration in coastal waters. *Nature* **235**, 158-160 (1972).

Barbier, E. B. et al. The value of estuarine and coastal ecosystem services. *Ecol. Monogr.* **81**, 169-193 (2011).

Bastida, F. et al. The active microbial diversity drives ecosystem multifunctionality and is physiologically related to carbon availability in Mediterranean semi-arid soils. *Mol. Ecol.* **25**, 4660-4673 (2016).

Byrnes, J. E., Roger, F. & Bagchi, R. Understandable multifunctionality measures using Hill numbers. Oikos, e09402 (2023).

Christianen, M. J. et al. Seagrass ecosystem multifunctionality under the rise of a flagship marine megaherbivore. *Glob. Chang. Biol.* **29**, 215-230 (2022).

Craft, C. et al. Forecasting the effects of accelerated sea-level rise on tidal marsh ecosystem services. *Front. Ecol. Environ.* **7**, 73-78 (2009).

Edgar, R. C. UPARSE: Highly accurate OTU sequences from microbial amplicon reads. *Nat. Methods* **10**, 996-998 (2013).

Garland, G. et al. A closer look at the functions behind ecosystem multifunctionality: A review. *J. Ecol.* **109**, 600-613 (2021).

Geurts, J. J. et al. Nutrient removal potential and biomass production by *Phragmites australis* and Typha latifolia on European rewetted peat and mineral soils. *Sci. Total. Environ.* **747**,141102 (2020).

Hensel, M. J. S. & Silliman, B. R. Consumer diversity across kingdoms supports multiple

functions in a coastal ecosystem. *Proc. Natl. Acad. Sci. USA.* **110**, 20621-20626 (2013).

Hemond, H. F., Nuttle, W. K., Burke, R. W. & Stolzenbach, K. D. Surface infiltration in salt marshes: Theory, measurement, and biogeochemical implications. *Water Resour. Res.* **20**, 591-600 (1984).

He, M., Wang, Z. & Tang, H. The chemical, toxicological and ecological studies in assessing the heavy metal pollution in Le An River, China. *Water Res.* **32**, 510-518 (1998).

He, Q., Altieri, A. H. & Cui, B. Herbivory drives zonation of stress-tolerant marsh plants. *Ecology* **96**, 1318-1328 (2015).

He, Q., Silliman, B. R., Liu, Z. & Cui, B. Natural enemies govern ecosystem resilience in the face of extreme droughts. *Ecol. Lett.* **20**, 194-201 (2017).

He, Q., Silliman, B. R., van de Koppel, J., & Cui, B. Weather fluctuations affect the impact of consumers on vegetation recovery following a catastrophic die-off. *Ecology* **100**, e02559 (2019).

Kowalska, J. et al. Soil pollution indices conditioned by medieval metallurgical activity-A case study from Krakow (Poland). *Environ. Pollut.* **218**, 1023-1036 (2016).

Liu, C., Zhao, C., Wang, A., Guo, Y. & Lee, D. J. Denitrifying sulfide removal process on high-salinity wastewaters. *Appl. Microbiol. Biotechnol.* **99**, 6463-6469 (2015).

Lozano, Y. M. et al. Effects of microplastics and drought on soil ecosystem functions and multifunctionality. *J. Appl. Ecol.* **58**, 988-996 (2021).

Magoč, T. & Salzberg, S. L. FLASH: Fast length adjustment of short reads to improve genome assemblies. *Bioinformatics* **27**, 2957-2963 (2011).

Martínez-Guijarro, R., Paches, M., Romero, I. & Aguado, D. Enrichment and contamination level of trace metals in the Mediterranean marine sediments of Spain. Sci. Total. Environ. **693**, 133566 (2019).

Michaletz, S. T., Cheng, D., Kerkhoff, A. J. & Enquist, B. J. Convergence of terrestrial plant production across global climate gradients. *Nature* **512**, 39-43 (2014).

Ollion, J., Cochennec, J., Loll, F., Escudé, C.& Boudier T. TANGO: A generic tool for high-throughput 3D image analysis for studying nuclear organization. *Bioinformatics* **29**, 1840-1841 (2013.).

Ramus, A. P., Silliman, B. R., Thomsen, M. S. & Long, Z. T., 2017. An invasive foundation species enhances multifunctionality in a coastal ecosystem. *Proc. Natl. Acad. Sci. USA* **114**, 8580-8585 (2017).

Rattan, L. Carbon sequestration. *Proc. R. Soc. B. Biol. Sci.* **363**, 815-830 (2008).

Risch, A. C. et al. Soil net nitrogen mineralisation across global grasslands. *Nat. Commun.* **10**, 4981 (2019).

Schimel, J. P. & Bennett, J. Nitrogen mineralization: Challenges of a changing paradigm. *Ecology* **85**, 591-602 (2004).

Schlesinger, W. H. Carbon sequestration in soils. *Science* **284**, 2095-2095 (1999).

Stackebrandt, E. & Goebel, B. M., 1994. Taxonomic note: A place for DNA-DNA reassociation and 16S rRNA sequence analysis in the present species definition in bacteriology. *Int. J. Syst. Evol. Microbiol.* **44**, 846-849 (1994).

Studds, C. E. et al. Rapid population decline in migratory shorebirds relying on Yellow Sea tidal mudflats as stopover sites. Nat. Commun. **8**, 14895 (2017).

Thomsen, M. S., Ramus, A. P., Long, Z. T. & Silliman, B. R. A seaweed increases ecosystem multifunctionality when invading bare mudflats. Biol. *Invasions* **21**, 27-36 (2019).

Xu X. et al. Native herbivores indirectly facilitate the growth of invasive Spartina in a eutrophic saltmarsh. *Ecology* **103**, e3610 (2022).

Zhang L.et al. Interactive effects of crab herbivory and spring drought on a *Phragmites australis*-dominated salt marsh in the Yellow River Delta. *Sci. Total. Environ.* **766**, 144254 (2021).

Zhang, W., Shen, J. & Wang, J. Linking pollution to biodiversity and ecosystem multifunctionality across benthic-pelagic habitats of a large eutrophic lake: A whole-ecosystem perspective. *Environ. Pollut.* **285**, 117501 (2021).

Zhang, X. et al. Macrophytes and crabs affect nitrogen transformations in salt marshes of the Yangtze River Estuary. Estuar. *Coast. Shelf Sci.* **225**, 106242 (2019).

Zhang, Y., Ji, G., Wang, C., Zhang, X. & Xu, M. Importance of denitrification driven by the relative abundances of microbial communities in coastal wetlands. *Environ. Pollut.* **244**, 47-54 (2019).

Yan X. et al. Heavy metals uptake and translocation of typical wetland plants and their ecological effects on the coastal soil of a contaminated bay in Northeast China. *Sci. Total. Environ.* **803**, 149871 (2022).

Yin, S., Bai, J., Wang, X., Wang, X., Zhang, G., Jia, J., Li, X. & Liu, X. Hydrological connectivity and herbivores control the autochthonous producers of coastal salt marshes. *Mar. Pollut. Bull.* **160**, 111638 (2020).
