# Supplementary material for: Shorebirds-driven trophic cascade helps restore coastal wetland multifunctionality
Source: Nat Commun. 2023 Dec 6;14:8076. doi: 10.1038/s41467-023-43951-3 (PMC10700615; doi:10.1038/s41467-023-43951-3)
Supplement: Supplementary file 3 — Description of Additional Supplementary Files [file 41467_2023_43951_MOESM3_ESM.pdf]

Description of Additional Supplementary Files

File Name: Supplementary Movie 1
Description: **Crabs intensively grazed on planted *Scirpus*, often during night, and nearly eliminated all planted *Scirpus* over just a few nights.** The movie shows photographs automatically taken using infrared cameras over five nights (19:00-05:00) following planting. Credit: Chunming Li.
